# The Versatility of SMRT Sequencing

**DOI:** 10.3390/genes10010024

**Published:** 2019-01-04

**Authors:** Matthew S. Hestand, Adam Ameur

**Affiliations:** 1 Division of Human Genetics, Cincinnati Children’s Hospital Medical Center, Cincinnati, OH 45202, USA; 2 Department of Pediatrics, University of Cincinnati College of Medicine, Cincinnati, OH 45202, USA; 3 Department of Immunology, Genetics and Pathology, Uppsala University, Science for Life Laboratory, 75025 Uppsala, Sweden; 4 Department of Epidemiology and Preventive Medicine, Monash University, Melbourne 32901, Australia

The adoption of single molecule real-time (SMRT) sequencing [1] is becoming widespread, not only in basic science, but also in more applied areas such as agricultural, environmental, and medical research. SMRT sequencing offers important advantages over current short-read DNA sequencing technologies, including exceptionally long read lengths (20 kb or more), unparalleled consensus accuracy, and the ability to sequence native, non-amplified, DNA molecules. These sequencing characteristics enable creation of highly accurate de novo genome assemblies, characterization of complex structural variation, direct characterization of nucleotide base modifications, full-length RNA isoform sequencing, phasing of genetic variants, low frequency mutation detection, and clonal evolution determination [2,3]. This Special Issue of Genes is a collection of articles showcasing the latest developments and the breadth of applications enabled by SMRT sequencing technology.

In basic science, SMRT sequencing enables studies into the molecular mechanisms of living cells at a new level of resolution. Perhaps the most advantageous feature of SMRT sequencing is that it facilitates sequencing of long DNA molecules at a very high accuracy. This has enabled the construction of high-quality reference genomes for a wide range of species, including new human genome assemblies, as presented in this special issue [4]. In addition, when SMRT sequencing is performed on native non-amplified DNA molecules, it is possible to access several layers of additional information hidden in the kinetic signals emitted by the polymerases during the sequencing reaction [1]. This kinetic information has been used to detect epigenetic modifications at base pair resolution and even phasing of methylation signatures in diploid organisms, as presented in this special issue [5]. Several important discoveries have already been made from this kinetic information, such as the widespread presence of 6mA modifications in the human genome [6], a modification that was previously thought to only be present in bacterial genomes. In addition to base modifications, SMRT sequencing data also enables us to study other events, such as DNA conformations [7]. Another aspect of SMRT sequencing is that it can be used to study RNA, and it is currently the only technology that can generate high-quality continuous sequences for full-length transcripts up to 10 kb or more. This makes it possible to study splicing variation at a completely new level of resolution [8,9]. SMRT sequencing is also paving the way for a new generation of computational approaches to explore and interpret these rich datasets [10,11,12]. In summary, SMRT sequencing is enhancing and even opening up new areas of basic research that were not accessible with previous sequencing technologies.

In terms of more applied areas, agriculture is benefiting from the advent of SMRT sequencing for examining important microbes, plants, and animals. SMRT sequencing, often with complementary technologies, has produced new genome assemblies for important crops, such as apples, maize, wine grapes, coffee, rice, black raspberries, asparagus, and cotton [11,13,14,15,16,17,18,19,20]. SMRT transcriptome sequencing has also given new insights into gene structures for rice, wheat, maize, sorghum, barley, and cotton [18,21,22,23,24,25]. Besides providing new references, these projects will improve plant cultivation, such as identifying drought and disease resistant genes. Strategies to detect genetically modified organisms (GMOs) have also been proposed and enhanced with SMRT sequencing [26]. Animal genome assemblies have been produced for several agriculturally valuable species, such as the horse, cow, goat, chicken (including its transcriptome), and commercially important fish like haddock and cod [27,28,29,30,31,32,33]. These will lead to improvements in animal breeding, management, and disease resistance. Finally, sequencing of pathogenic bacteria and fungi affecting agriculturally important species is providing insight into the diversity and virulence factors of these pathogens, which in turn will assist in disease risk and management [34,35,36].

In environmental research, systematic efforts are ongoing to generate reference sequences for thousands of bacterial strains and microorganisms. Recently, this has expanded to the genomes of larger organisms, including vertebrates [37]. SMRT sequencing can also play an important role in ecology research, such as monitoring the composition of fungi in environmental soil or water samples [38,39]. New high-quality references for animal genomes, such as the great apes [40], will provide an invaluable resource for future evolutionary studies. During the last few years, new genome assemblies have also been created for several endangered species, including Hawaii’s last crow species [41], aiding in conservation efforts.

Though SMRT sequencing has primarily been applied to basic research, there is a growing implementation for clinical utility [3,42]. The long and highly accurate reads produced from SMRT sequencing have proven to be useful to resolve complex and repetitive regions of the human genome associated with disease. SMRT sequencing is also a sensitive method to detect minor variants in cancer and infectious disease. Although most current methods are based on targeted sequencing, the value of long reads is also becoming apparent for whole-genome sequencing, which allows clinical professionals to resolve repeat expansions, transposable element insertions, and other complex genomic rearrangements that are difficult or even impossible to assess using short-read sequence data.

As we look forward, this technology will provide even longer and more accurate reads at a higher throughput. This will enable routine de novo assembly of both alleles in large diploid genomes, accompanied with tissue specific epigenetic DNA modification information. As a consequence, there will be a demand for a new generation of computational tools to compare complete genomes to each other, as opposed to a reference standard, and to phase genetic variants and epigenetic modifications over large chromosomal regions. By sequencing thousands of individuals with long reads, it will be possible to obtain a detailed picture of complex structural variation within large population cohorts of humans, as well as for other species. Such endeavors will give new insights to the function of the repetitive parts of the genome, and likely explain the cause of many genomic diseases. Looking further on the horizon, SMRT sequencing can be envisioned in combination with other technical advances, such as single cell sequencing to provide information on the epigenetic modifications occurring in single cells. SMRT sequencing has been steadily evolving since the commercial introduction of the technology in 2011. Just as short-read technologies have replaced microarrays and Sanger sequencing for a host of applications, we envision long-read single-molecule sequencing to replace short-read platforms for a majority of applications, as well as continue to evolve into new applications, throughout many different areas in the coming decade.
